# SG-APSIC1201: Knowledge and awareness of healthcare workers in a residential care home regarding the use of personal protective equipment (PPE) during the COVID-19 pandemic: A pilot study

**DOI:** 10.1017/ash.2023.22

**Published:** 2023-03-16

**Authors:** Cho Yan Chung, Chau Ho Yin, Chu Hoi Fung, Chung Cho Yan, Fong Long Kit, Foo Siu Fung, Ho Tsz Yan, Or Pui Lai Peggy, Ching Tai Yin Patricia

**Affiliations:** Education University of Hong Kong, Hong Kong, China; Department of Health and Physical Education, The Education University of Hong Kong, Hong Kong, China; Department of Health and Physical Education, The Education University of Hong Kong, Hong Kong, China; Department of Health and Physical Education, The Education University of Hong Kong, Hong Kong, China; Department of Health and Physical Education, The Education University of Hong Kong, Hong Kong, China; Department of Health and Physical Education, The Education University of Hong Kong, Hong Kong, China; Department of Health and Physical Education, The Education University of Hong Kong, Hong Kong, China; Department of Health and Physical Education, The Education University of Hong Kong, HongKong, China; WHO Collaboration Centre for Infectious Disease Epidemiology and Control, School of Public Health, The University of Hong Kong, Hong Kong, China

## Abstract

**Background:** According to the World Health Organization (WHO), as of April 9, 2022, there had been 494,587,638 confirmed COVID-19 cases and 6,170,283 deaths reported worldwide. In Hong Kong, in recent outbreak, ~55% of confirmed cases were residential care home (RCH) residents and >800 staff were infected. In 2016, ~15% of people aged ≥80 years were living in residential care homes. **Objectives:** To assess healthcare worker (HCW) knowledge level and attitudes about PPE use in residential care homes. **Methods:** This cross-sectional study, included participants who worked in the residential care homes, registered as healthcare workers (HCWs). HCWs who were part-time staff or worked <3 months in the residential care home were excluded. Ethical review approval from the faculty research committee of the university was obtained in January 2022. The Knowledge, Attitude, Practical (KAP) questionnaire was adapted. The questionnaire has 33 items pertaining to knowledge, attitude, and practice regarding PPE. **Results:** In total, 50 questionnaires were received; 32 respondents (64%) were female and 18 (36%) were male. Nearly half of the participants had completed a high diploma course, and 32% had graduated from secondary school. Using ANOVA, there were no significant differences of education level of participants or participant knowledge level of PPE [F(2,47) = .181; *P* = .835], attitudes [F(2,47) = 1.995; *P* = .147] and practice [F(2,47) = .459; *P* = .635]. The Pearson correlation was used to measure the relationship between knowledge level and PPE practices. Our results indicated a significant difference and moderate correlation between knowledge level and PPE practice among HCWs. **Conclusions:** Knowledge level does not directly affect HCW practice regarding PPE. PPE practice skills have been influenced by various factors during the pandemic situation, such as availability of PPE, manpower, workload, and communication.

